# Tendon Regeneration and Repair with Stem Cells

**DOI:** 10.1155/2012/316281

**Published:** 2011-10-27

**Authors:** S. MacLean, W. S. Khan, A. A. Malik, M. Snow, S. Anand

**Affiliations:** ^1^University of Manchester, Manchester M13 9PL, UK; ^2^Royal National Orthopaedic Hospital, Middlesex HA7 4LP, UK; ^3^Royal Orthopaedic Hospital, Birmingham B31 2AP, UK

## Abstract

The use of stems cells in tendon repair is of particular interest given the frequency of tendon injuries worldwide together with the technical difficulty often encountered when repairing or augmenting tendons. Stems cells have the capability to differentiate into a variety of different cell types including osteocytes and tenocytes, and if normal architecture of damaged tendon (either macroscopic or microscopic) could be restored, this would significantly improve the management of patients with these injuries. There is already encouraging research on the use of stems cells clinically although considerable further work is required to improve knowledge and clinical applications of stem cells in tissue engineering.

## 1. Anatomy and Pathophysiology of Tendon Damage

Tendons attach muscle to bone and function to transmit tensile loads from muscle to bone and to enable the muscle belly to be at an optimal distance from the joint. The microstructural composition is approximately 20% cellular (fibroblasts secreting collagen) and 80% extracellular matrix. The extracellular matrix consists of mainly water, collagen, and ground substance [1].

More than 90% of collagen in tendons is type 1 with the remainder being type 3. These molecules are aligned in parallel to form microfibrils, which are further aggregated to form bundles [1]. This allows them to handle high unidirectional tensile loads. Ground substance consists mainly of proteoglycans, glycoproteins, and plasma proteins. These bind the extracellular water in the tendon, helping to stabilise the collagenous skeleton and contributing to the overall tendon strength. Elastin, secreted by fibroblasts, forms highly cross-linked sheets, allowing the tendon to stretch and coil, contributing to tissue recovery after loading.

The tendinous zone of insertion (enthesis) is a progressive structural change from tendon to bone, resulting in increased stiffness and decreased stress concentration. It is often the site of tendinopathic change and injury. It is divided into four zones; parallel collagen fibres at the end of tendon, unmineralised fibrocartilage, and mineralised fibrocartilage, which merges into cortical bone [1].

The blood supply to tendons is inferior to that of most other connective tissues. The blood supply is through a sparse supply of arterioles or a vincula (mesotenon) and subsequent diffusion through the tendon substance. They have a low metabolic rate. Both these factors have implications for the intrinsic healing potential of the tissue [1].

There are a number of factors affecting the biomechanical properties of tendons and subsequent tendinosis (degenerative tendon) or tendinopathy (an inflammatory reaction secondary to rupture or vascular damage) [2]. Ageing results in a decrease in collagen diameter and number. Endocrine factors play a part, and pregnancy is associated with a decreased stiffness of pelvic tendons. Pharmacological agents such as corticosteroids and anabolic steroids are associated with tendon rupture. Systemic disease and genetics affect the intrinsic healing potential. Repetitive microtrauma and fatigue failure often leads to calcification and an inflammatory reaction. Macrotrauma results in the acute rupture of a tendon due to a force above the ultimate tensile strength, either at the tendinous insertion onto bone or in the tendon substance itself.

The healing response is variable and usually poor. There is an initial rapid haemorrhagic and inflammatory phase. This is followed by a proliferative phase, with fibroblast production of new matrix. Remodelling occurs several weeks after injury consisting of maturation and orientation of collagen fibres. Although there are external influences affecting tendon repair which can be controlled, there are intrinsic metabolic limitations to healing. Often a surgical solution is necessary to repair or reconstruct tendon. Rotator cuff tears and subsequent repair illustrate the magnitude of the problem. Rotator cuff injuries accounted for 4.4 million outpatient appointments in the US in 2003 [3]. It is estimated that at least 13% of individuals between the ages of 50 and 59 and 51% of people over the age of 80 experience rotator cuff injuries [4], and over 50,000 patients in the US require direct repair each year [5]. Despite this, repair can fail up to 40% of the time, leading to impaired shoulder biomechanics and subsequent weakness and degenerative osteoarthritic changes [6]. Tissue engineering and the use of stem cells has sought to provide a solution to this common cause of musculoskeletal morbidity.

## 2. Stem-Cell Potential

Stem cells may be totipotent, pluripotent, or multipotent, depending on tissue type. Totipotent cells form all the cells and tissues that contribute to the formation of an organism. Only the embryo itself is totipotent. Pluripotent stem cells (PSCs) can form most cells of an organism from all three germ cell layers. Embryonic stem cells present in the fertilised oocyte, zygote, and morula [7]. Pluripotent cells have the ability to expand in vitro almost indefinitely and form tissues from ectoderm, mesoderm, or endoderm. There are concerns about tumour formation in vivo and major ethical concerns, however, which have thus far restricted their use.

Multipotent cells form a number of cells or tissues that are usually restricted to a particular germ layer. Multipotent cells are derived from specific tissue compartments in the adult. The two main types of multipotent stem cell are haemopoietic and mesenchymal type, and both are usually derived from adult bone marrow, but occasionally from fat, skin, periosteum, and muscle. Mesenchymal stem cells (MSCs) are multipotent, capable of differentiating into several connective tissue types including osteocytes, chondrocytes, adipocytes, tenocytes, and myoblasts [8]. Mesenchymal stem cells have the advantage of being easily obtainable in adult tissue and, with the appropriate microenvironment can differentiate into various target tissue types. 

Research on tendon healing and the use of stem cells has thus far been limited to animal studies, with the majority using mesenchymal stem cells. 

### 2.1. Mesenchymal Stem Cells

MSCs can arise from a number of sources as already highlighted. Kryger et al. [9] isolated tenocytes, sheath fibroblasts, bone-marrow-derived stem cells, and adipose stem cells from adult rabbits and used them in a flexor tendon model. Although adipose-derived stem cells proliferated faster in culture, at six weeks, there was no difference with regards to cell viability, senescence, or collagen expression.

Tempfer et al. [10] examined biopsies of intact human supraspinatus tendons and showed that stem cell tendon precursors (tenocytes) were present in the tissue. Pryce et al. [11] showed that TGF beta signalling may play an important role in the recruitment of tenocytes. Mazzocca et al. [12] aspirated bone marrow from the bone anchor tunnel in the humeral head during arthroscopic rotator cuff repair in 23 patients. Using a novel device, in the operating room, stem cells were isolated from this aspirate and their presence and osteogenic potential confirmed. This study showed that stem cell-rich bone marrow is exposed following arthroscopic drilling of the humeral head. These stem cells harbour potential to differentiate into osteoblasts and tenocytes to regenerate the bone-tendon interface. Novel solutions for the recruitment and activation of these cells in combination with growth factors, gene therapy and an appropriate scaffold may provide improved strength of the rotator cuff following surgical repair.

Awad et al. [13] showed that there was a significant improvement in tendon repair when MSCs were injected into patellar tendon defects in rabbits. Compared to a cell-free collagen control at four weeks, MSC-mediated repair tissue demonstrated significant increases in stress, modulus, and strain energy density of 26%, 18%, and 33%, respectively. Chong et al. [14] examined the histology and modulus of Achilles tendon defects in rabbits over a 12-week period. Compared to controls, it was shown that at three weeks, there was an improvement in collagen organisation and modulus in the MSC group. By 12 weeks, however, this difference was insignificant, suggesting that MSCs may improve tendon healing in the early stages only.

The tendon-bone interface is a common site of rupture, especially at repetitive low-loading forces, for example, in rotator cuff tendinopathy. Chang et al. [15] examined healing potential of infraspinatus tendon in rabbits at the tendinous insertion using a periosteal graft containing autologous MSCs. Histological examination from 4 to 12 weeks showed gradual progression in healing from fibrotic tissue to mineralised fibrocartilage. There was an associated significant increase in failure load with time compared to controls. Ju et al. [16] used Achilles tendon grafts in a rat anterior cruciate ligament model (ACL). He undertook an ACL reconstruction and then injected the tibiofemoral bone tunnel with MSCs. Tendon-bone analysis at 2 weeks showed the proportion of collagen fibres at the interface tissue was significantly higher in the MSC group compared to controls. At 4 weeks in both groups, the implanted tendon appeared to attach directly to bone. The benefit of injecting MSCs, therefore, may give early benefit in this model. A study by Nourissat et al. [17] evaluated healing at the tendon-bone interface at the Achilles tendon in a rat model. After the tendon-bone interface was destroyed, the tendon was either left to heal, or an injection was given of chondrocytes or MSCs. At 45 days, it was found that cell injection of either chondrocytes or MSCs significantly improved healing compared to controls left to heal without an injection. A new enthesis was produced in the injection groups but not in controls, and in only the MSC group was this organised as in normal enthesis tissue.

The treatment of tendonitis by stem cells was studied by Lacitignola et al. [18] in horses. Tendonitis was induced by an injection of collagenase into the superficial flexor tendons. Three weeks later, bone marrow mesenchymal cells, bone marrow mononucleated cells, or controls of fibrin were injected into the tendons. In the stem cell-treated groups, there was significantly improved healing histologically with a higher collagen type 1 to type 3 ratio and improved fibre orientation compared to controls. Another equine flexor tendon model for tendonitis compared MSCs, MSCs with insulin-like growth factor-1 (IGF-1) gene-enhanced MSCs, and controls. Both IGF-1 MSCs and MSC groups showed significantly improved tendon histology at 8 weeks compared to controls [19].

### 2.2. Pluripotent Stem Cells

Watts et al. [20] reported on the use of an injection of foetal-derived embryonic-like stem cells for superficial flexor tendon injuries in horses. Compared to controls at eight weeks, there was no significant difference in tendon matrix gene expression, proteoglycan, collagen, or DNA content between the tendons. There was, however, improved tissue architecture, tendon size, lesion size, and linear fibre pattern in the lesions treated with stem cells. The tensile strengths of the healing tendons were not tested, however.

Turner et al. [21] reviewed the use of amniotic stem cells for the engineering of a diaphragmatic tendon graft in newborn lambs. Failure rate was higher in the control group (acellular prosthetic graft). Tensile strength testing and collagen levels were significantly higher in the grafts containing stem cells.

The use of stem cell-coated sutures could have obvious theoretical benefits in surgical repair of tendons. Yao et al. [22] evaluated the fats of pluripotential embryonic stem cells seeded to a suture carrier in acellularised, sectioned rabbit Achilles tendon. At day 5, fluorescence under microscopy showed live metabolically active pluripotential cells at the tendon repair site. The same author showed that cell adherence at seven days was greater in FibreWire sutures when first coated with poly-1-lysine or fibronectin [23].

Guest et al. [24] examined the difference between MSCs and embryo-derived PSCs injected into damaged superficial digital flexor tendons in horses. At 90 days following injection, there had no signs of immune reaction to the allogenic PSCs and no sign of tumour formation. Survival rate was greater, with PSCs maintaining a constant level over 90 days in contrast to MSCs which showed less than 5% survival over ten days and a subsequent decline thereafter. PSCs also showed an ability to migrate to other areas of damaged tendon in contrast to MSCs.

### 2.3. Tissue Engineering for Tendon Regeneration

There are now several studies illustrating the potential for the use of stem cells not only in tendon repair, but also other their use in other tissue engineering applications [25–29]. Several studies have shown that a mechanical stimulus improves tendon healing. It has been shown in patellar tendon defects in rabbits that two weeks of in vitro mechanical stimulation significantly increased collagen type 1 and collagen type 3 gene expression of stem cell-collagen sponge constructs. These constructs exhibited 2.5 times increased linear stiffness and 4 times the linear modulus of controls [30]. The degree of mechanical loading has been shown to affect cell differentiation. One study showed that low mechanical in vitro stretching of MSCs into tenocytes, where as larger stretching at 8% induced differentiation into adipocytes, chondrocytes, and osteocytes [31]. In clinical practise, lipid accumulation and calcification in a healing tendon may lead to pain and a detrimental functional outcome. 

In vivo, the extracellular matrix of tendon provides fibroblasts with the architecture to support development and function. During tissue engineering, therefore, a scaffold is needed to mimic this matrix. The optimal cell: matrix ratio to support tendon function is debated. Juncosa-Melvin et al. [32] examined cell: collagen ratios in Achilles tendon defects in rabbits. It was shown that constructs with a lower cell density at 12 weeks achieved higher stiffness and modulus values. Nirmalanandhan et al. [33] showed that above a threshold value of cell density, percentage reductions in collagen concentration influence contraction kinetics more than equivalent percentage increases in cell seeding density. The alignment of stem cells on scaffolds may be important. Yin et al. [34] showed that foetal stem cells placed in randomly oriented scaffolds in vitro led to osteogenic differentiation. In contrast, aligned nanofibres induced the formation of spindle-shaped cells and tendon-like tissue. Obviously, controlling scaffold conditions is vital to the effective differentiation of these cells and the ultimate mechanical properties of the healing tissue.

Butler et al. [35] found that in rabbits with patellar tendon defects, there appeared to be 4 important factors which improved the biomechanical properties of the healing tendon. Replacing the suture with end posts in culture and lowering the MSC concentration in cell-scaffold constructs resulted in failure forces greater than peak in vivo forces that were measured for all activities and tangential stiffness similar to normal tendon. Augmenting the scaffold gel with a type 1 collagen sponge increased repair stiffness, and mechanically stimulating these constructs further improved biomechanics in the healed tendon. 

#### 2.3.1. Use of Growth Factors in Tissue Engineering

Recently, Gulotta et al. [36] has highlighted the importance of gene expression in stem cells for tendon healing. In a rat supraspinatus model, MSCs after injection were present and metabolically active, but no difference in the biomechanical strength of the repairs, the cross-sectional area, peak stress to failure, or stiffness compared to controls could be found. A further study compared an MSC group and a group who had received adenoviral MT1 matrix metalloproteinase-transduced MSCs (Ad-MT1-MMP). Although no difference was found at 2 weeks, at 4 weeks, the Ad-MT1-MMP group had significantly more fibrocartilage, higher load to failure, stress to failure, and stiffness values as compared to MSCs [37]. It has also been shown that MSCs expressing BMP-2 and the transcription factor Smad8ca lead to differentiation into a tenocyte lineage [38]. It has been proposed that expression of Smad8ca lead to the production of MMPs. Shahab-Osterloh et al. [39] showed that MSCs with adenoviral-induced Smad8ca and BMP-2 exhibit both tendinous and osteogenic properties in mice and can aid formation, therefore, of bone-tendon interface. Numerous other studies highlight the beneficial effect of BMP in tendon-bone interface healing [40].

The quality of tendon that forms from bioengineering may be a concern still, with ectopic bone formation being a problem in the healing tissue. Harris et al. [41] showed that this is likely related to alkaline phosphatase activity and may be higher in 3D in vitro constructs compared to tissue engineering on a 2D monolayer.

## 3. Summary

Tendon healing is limited by numerous intrinsic and extrinsic factors [42–47]. These have implications for the athlete in a “macrotrauma” acute rupture setting or in repetitive microtrauma leading to tendonitis. Like all connective tissues, tendon is vulnerable to the effects of ageing, inevitably leading to cell senescence of tenocytes, resulting in an extracellular matrix devoid of collagen and weakened tissue. The principles of tissue engineering involve a complex interplay of factors [48–52]. Local delivery of growth factors, the use of plasmids, and scaffolds are several. These in combination with stem cells or genetically modified stem cells have been shown to contribute to tendon healing in numerous animal studies [53–56]. Concerns arise as to tumour formation and immune reactions to allogenic sources, and there are obvious ethical considerations. The use of stem cells is a promising treatment in the armamentarium of the physician or surgeon, but further research is needed to decide on the optimal strategy in humans.
